# The 0.3-kb fragment containing the R-U5-5’leader sequence of Friend murine leukemia virus influences the level of protein expression from spliced mRNA

**DOI:** 10.1186/1743-422X-10-124

**Published:** 2013-04-19

**Authors:** Yeng Cheng Choo, Yohei Seki, Akihito Machinaga, Nobuo Ogita, Sayaka Takase-Yoden

**Affiliations:** 1 Department of Bioinformatics, Faculty of Engineering, Soka University, Hachioji, Tokyo 192-8577, Japan

**Keywords:** Retrovirus, Murine leukemia virus, R-U5, 5’leader sequence, Protein expression, Splicing, Post-transcriptional events

## Abstract

**Background:**

A neuropathogenic variant of Friend murine leukemia virus (Fr-MLV) clone A8 induces spongiform neurodegeneration when infected into neonatal rats. Studies with chimeras constructed from the A8 virus and the non-neuropathogenic Fr-MLV clone 57 identified a 0.3-kb KpnI-AatII fragment containing a R-U5-5’leader sequence as an important determinant for inducing spongiosis, in addition to the *env* gene of A8 as the primary determinant. This 0.3-kb fragment contains a 17-nucleotide difference between the A8 and 57 sequences. We previously showed that the 0.3-kb fragment influences expression levels of Env protein in both cultured cells and rat brain, but the corresponding molecular mechanisms are not well understood.

**Results:**

Studies with expression vectors constructed from the full-length proviral genome of Fr-MLV that incorporated the *luciferase* (*luc*) gene instead of the *env* gene found that the vector containing the A8-0.3-kb fragment yielded a larger amount of spliced *luc*-mRNA and showed higher expression of luciferase when compared to the vector containing the 57-0.3-kb fragment. The amount of total transcripts from the vectors, the poly (A) tail length of their mRNAs, and the nuclear-cytoplasm distribution of *luc*-mRNA in transfected cells were also evaluated. The 0.3-kb fragment did not influence transcription efficiency, mRNA polyadenylation or nuclear export of *luc*-mRNA. Mutational analyses were carried out to determine the importance of nucleotides that differ between the A8 and 57 sequences within the 0.3-kb fragment. In particular, seven nucleotides upstream of the 5’splice site (5’ss) were found to be important in regulating the level of protein expression from spliced messages. Interestingly, these nucleotides reside within the stem-loop structure that has been speculated to limit the recognition of 5’ss.

**Conclusions:**

The 0.3-kb fragment containing the R-U5-5’leader sequence of Fr-MLV influences the level of protein expression from the spliced-mRNA by regulating the splicing efficiency rather than transcription, nuclear export of spliced-mRNA, or poly (A) addition to mRNA. Seven nucleotides in the 0.3-kb fragment, which reside within the stem-loop structure that has been speculated to limit recognition of the 5’ss, could pinpoint the function of this region.

## Background

The simple retroviruses, including MLV, are characterized by a coding structure in which the *gag, pol* and *env* genes are flanked by two long terminal repeats (LTRs), a 5’LTR and 3’LTR. Proteins responsible for the constitution of the inner structures of the virion are encoded by the *gag* gene, which includes the matrix, capsid and nucleocapsid proteins. The *pol* gene encodes the enzymatic proteins, i.e. the reverse transcriptase, protease, integrase and RNase H and the *env* gene encodes the proteins protruding out from the viral particle surface, namely the surface (SU) and transmembrane (TM) proteins [1]. Transcription begins from the R region of the 5’LTR and ends at the polyadenylation signal located at the R region at the other end of the 3’LTR. A 5’ss is located in the 5’leader sequence and a 3’splice site (3’ss) is located at the 3’ end of the *pol* gene. Only a singly spliced mRNA is usually found in simple retroviruses. Gag and Pol proteins are translated from the unspliced full-length viral mRNA, and the Env protein is translated from the spliced *env*-mRNA [1]. In contrast, it has been reported that human immunodeficiency virus (HIV) type 1, which is a complex retrovirus, could generate up to 40 different spliced RNAs using four 5’ss and nine 3’ss [2-4].

A neuropathogenic variant of Fr-MLV, clone A8, induces spongiform neurodegeneration in neonatal rats. Studies with chimeras constructed from the A8 virus and the non-neuropathogenic Fr-MLV clone 57 identified a 0.3-kb KpnI-AatII fragment containing the R-U5-5’leader sequence as an important determinant of neuropathogenicity, in addition to the *env* gene of A8 as the primary determinant [5]. The A8-Env protein expression level is also correlated with neuropathogenicity [5,6]. Chimeric virus Rec5, which contains the A8-*env* gene on the background of 57, did not exhibit neuropathogenicity. In contrast, the chimeric virus R7f, which contains a 0.3-kb fragment of A8 and the A8-*env* gene on the background of 57, induced spongiform neurodegeneration. It has been shown that the expression level of Env protein in both R7f-infected cultured cells and in brains of R7f-infected rats was higher than in the Rec5-infected cultured cells and brains of Rec5-infected rats [5,6]. These findings suggested that the 0.3-kb fragment influences Env protein expression. However the steps of gene expression at which the 0.3-kb fragment may influence Env expression have yet to be elucidated.

Given that the 0.3-kb fragment containing the R-U5-5’leader sequence is the first untranslated region that exists in all variants of retroviral transcripts, this region dynamically impacts various stages of the viral life cycle. The R region, present at both ends of viral RNA, mediates the jump of reverse transcriptase from the 5’ site to the 3’ site during the synthesis of minus-strand DNA [7,8], possibly by mediating genome circularization [9-11]. In addition, the stem-loop structure of the R region is important for transcriptional activity and enhances gene expression of a variety of retroviruses, including HIV, human T cell leukemia virus, bovine leukemia virus, avian reticuloendotheliosis virus, MLV, mouse mammary tumor virus, human foamy virus, and spleen necrosis virus [12-24]. The end of the U5 region is marked by the beginning of the primer binding site (PBS) for reverse transcription [25-27]. The surrounding region of U5 with the 5’leader sequence (which extends from the PBS to the AUG codon of *gag*) has specific sequences with distinct secondary structure features [28,29]. There is strong evidence that this region is robust and that the secondary structures presented are fine-tuned to regulate one stage of RNA processes, and they could also act as inhibitors for other processes [30]. For example, the stem loop of DIS-1 (dimer initiation site-1), which plays a role in initiating viral RNA dimer formation, is situated immediately downstream of the 5’ss. By deleting this stem loop structure, the splicing efficiency of a modified Akv-MLV increased 5–10 fold, illustrating the modulating effect of DIS-1 on the production of viral genomes [31]. Interestingly, sequences upstream of 5’ss have also been reported to be limiting factors for splicing regulation [32]. A secondary structure known as the B monomer was presented in Mougel et al. [28] and is a discerning trait in the MLV. This secondary structure, which is adopted in the dimeric RNA form, has also been shown to limit the recognition of U1snRNA to the splice donor, thereby also regulating the viral RNA production volume. Finally, the highly dynamic encapsidation structure that has been studied extensively in the prototype of MLV, Moloney MLV (Mo-MLV) [33-35], is important for dimerization of the genomic RNA [36,37]. It includes an IRES (internal ribosomal entry segment) [38,39] and also functions in the transport of viral intron-containing RNAs from the nucleus to the cytoplasm [34,40].

In this study, to investigate the role of the 0.3-kb fragment containing the R-U5-5’leader sequence in the expression of Env protein of Fr-MLV, we constructed expression vectors having the full-length proviral genome of Fr-MLV with the *luciferase* (*luc*) gene incorporated in place of the *env* gene. We then examined the effects of the 0.3-kb fragment on several steps affecting protein expression levels in NIH3T3 cells. The results showed that the 0.3-kb fragment of A8 enhanced protein expression levels from the spliced mRNA through up-regulating the efficiency of splicing compared with the 0.3-kb fragment of 57, rather than through increased transcription, poly (A) addition to mRNA, or nuclear export of spliced mRNA. Furthermore, we investigated more specifically the roles of the nucleotides that differ between A8 and 57 sequences in defining the function of the 0.3-kb fragment. Lastly, we discuss the possible mechanism by which the 0.3-kb fragment participates in protein expression.

## Results

### The 0.3-kb fragment effects on luciferase protein expression and the amount of spliced *luc*-mRNA

**Figure 1:**
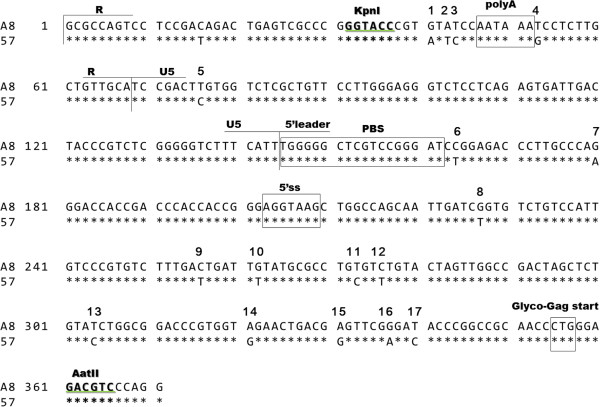
**Alignment of the 0.3-kb KpnI-AatII fragment of A8 [accession no. D88386] and 57 [accession no. X02794].** Asterisks represent the sequence identity. PolyA: polyadenylation signal; PBS: primer binding site; 5’ss: 5’ splice site; glyco-Gag start; the start codon of glycosylated-Gag protein. Nucleotides that differ between A8 and 57 within the 0.3-kb fragment are numbered.

**Figure 2:**
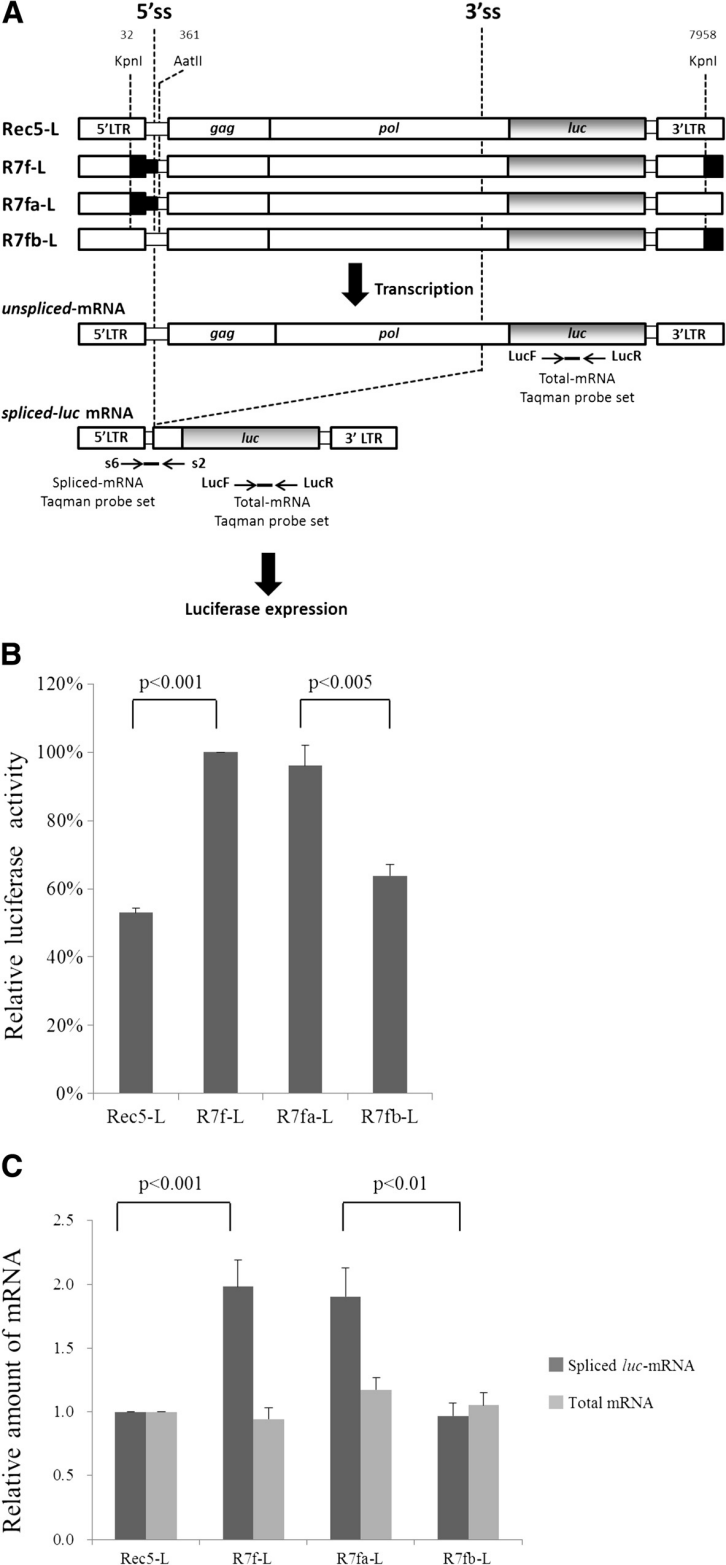
**Structures of luciferase expression vectors (A).** In the viral genomes, solid regions are sequences derived from the A8 virus and open regions are sequence derived from the 57 virus. The numbering of nucleotides is based on the transcript. Vectors, primers and probes used to detect the corresponding mRNA by RT-PCR are indicated on the vectors. 5’ss: 5’splice site; 3’ss: 3’splice site. Relative Luciferase activity (**B**) and relative amount of spliced *luc*-mRNA and total mRNA (**C**). The graphs show the mean values from 4–7 independent results and the SEM are indicated as half whiskers. The statistical comparison was carried out using the *t* test.

This study is based on a background study which revealed that the 0.3-kb KpnI-AatII fragment containing the R-U5-5’leader sequence was essential for the induction of spongiform neurodegeneration and for up-regulation of Env protein expression [5]. The purpose of the present study is to further investigate the function of the 0.3-kb fragment in retroviral gene expression. Between the A8 and 57 sequences within the 0.3-kb fragment, 17 nucleotides differ (Figure 1). In our previous study, neuropathogenic R7f, which contains the A8-0.3-kb fragment and the A8-*env* gene on the background of 57, was shown to increase Env expression about 3-fold compared to non-neuropathogenic Rec5, which contains the 57-0.3-kb fragment and the A8-*env* gene on the background of 57 [5,6]. To investigate the function of the 0.3-kb fragment in viral gene expression, the full-length viral genomes of Rec5 and R7f were recombined with the *luc* gene, in which the viral *env* gene was replaced with the *luc* gene to produce the luciferase expression vectors Rec5-L and R7f-L, respectively (Figure 2A). Both Rec5-L and R7f-L were constructed using the complete virus 57 sequences, however, in R7f-L the 0.3-kb fragment was derived from the viral A8 sequence. The luciferase protein is translated from spliced mRNA of these expression vectors. After transfection of the vectors into NIH3T3 cells, luciferase activities were measured. The luciferase activity of R7f-L increased by 2-fold compared to that of Rec5-L (p < 0.001) (Figure 2B). To determine the role of the 0.3-kb fragment positioned at the 5’LTR-leader sequence and the 3’LTR in the expression vectors, R7fa-L and R7fb-L were constructed (Figure 2A). R7fa-L, which carries the 0.3-kb fragment of A8 only at the 5’LTR-leader sequence, exhibited the same amount of luciferase activity as R7f-L, and the luciferase activity of R7fb-L, which carries the 0.3-kb fragment of A8 only at the 3’LTR, showed luciferase activity that was lower compared to R7fa-L (p < 0.005) and comparable to that of Rec5-L (Figure 2B).

Furthermore, the effect of the 0.3-kb fragment on the *luc*-mRNA level was also determined. The spliced *luc*-mRNA levels were measured by real-time RT-PCR using s6 and s2 primers (Figure 2A). These primers were designed to amplify a fragment containing the splicing junction region from the cDNA of spliced transcripts. The amount of spliced *luc*-mRNA from R7f-L increased by 2-fold compared to that from Rec5-L (p < 0.001) (Figure 2C). The amount of spliced *luc*-mRNA from R7fa-L was the same as that from R7f-L. The amount of spliced *luc*-mRNA from R7fb-L was lower than that from R7fa-L (p < 0.01) but was comparable with that from Rec5-L (Figure 2C). The amount of spliced mRNAs paralleled the luciferase activity. Next, to examine effects of the 0.3-kb fragment on transcriptional activity, the amount of total transcripts from expression vectors were measured by real-time RT-PCR using the LucF and LucR primers (Figure 2A). The amounts of total mRNA measured for all of the expression vectors were comparable (Figure 2C).

### The 0.3-kb fragment did not affect the poly (A) tail length of mRNA or the nuclear-cytoplasmic distribution of *luc*-mRNA

**Figure 3:**
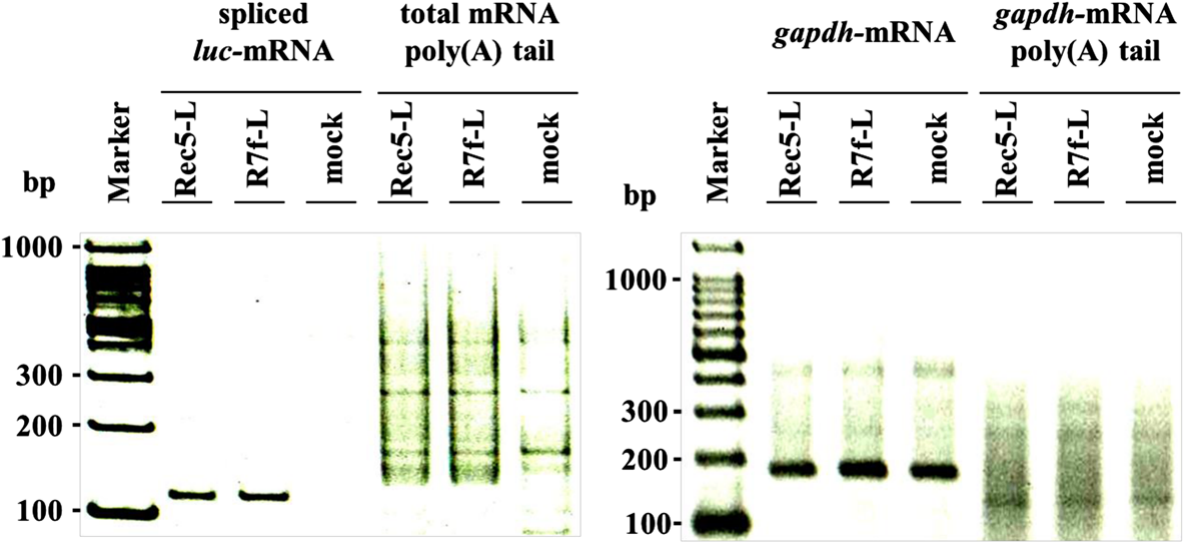
**Determination of poly (A) tail length.** Total RNAs extracted from 24 hours post-transfected Hela-cells were ligated with the anchor primer RVP3 oligo. First strand cDNA synthesis was carried with an antisense sequence to the anchor primer. To detect poly (**A**) tail length, the transcripts derived from Rec5-L and R7f-L were then selectively amplified using the forward primer for viral U3 sequences of the 3’ LTR and the reverse primer for the RVP3 sequence. To confirm that the first strand of cDNA that was synthesized with an anti-RVP3 oligo strand contained spliced luciferase-mRNA, PCR was performed using the primer set of f-597 and s2. As a control, the *gapdh*-mRNA and poly (A) tail length of *gapdh*-mRNA were detected in Rec5-L and R7f-L transfected cells. The PCR products were electrophoresed and visualized by ethidium-bromide staining.

In general, the poly (A) tail length of mRNA is correlated with the efficiency of translation. Therefore, to examine whether or not the 0.3-kb fragment influences polyadenylation of viral mRNA, the poly (A) tail lengths of mRNA from Rec5-L and R7f-L transfected Hela cells were compared. Total RNA was harvested and anchored with the RVP3 primer before the first strand of cDNA was synthesized with an anti-RVP3 oligo strand. To determine the poly (A) tail length, the transcripts derived from Rec5-L and R7f-L were selectively amplified using the forward primer for viral U3 sequences of the 3’LTR and the reverse primer for the RVP3 sequence. PCR products viewed on electrophoresed gels showed no detectable differences in the smeared patterns indicating the poly (A) tail lengths of transcripts derived from R7f-L and Rec5-L (Figure 3). In this system, the poly (A) tail lengths of transcripts containing both the unspliced mRNA and the spliced mRNA derived from the vectors could be detected. Therefore, to confirm that the first strand of cDNA synthesized with an anti-RVP3 oligo strand contained spliced-mRNA, from which luciferase protein was translated, PCR was performed using the primer set of f-597 and s2. As shown in Figure 3, a 113-bp band that came from spliced-mRNA was detected in both Rec5-L and R7f-L transfected cells. As a control, the poly (A) tail length of *gapdh*-mRNA was examined in Rec5-L and R7f-L transfected cells. In both of these cells, a 177-bp band for *gapdh*-mRNA was detected, and there were no detectable differences in the smeared patterns indicating the poly (A) tail length of *gapdh*-mRNA (Figure 3).

**Figure 4:**
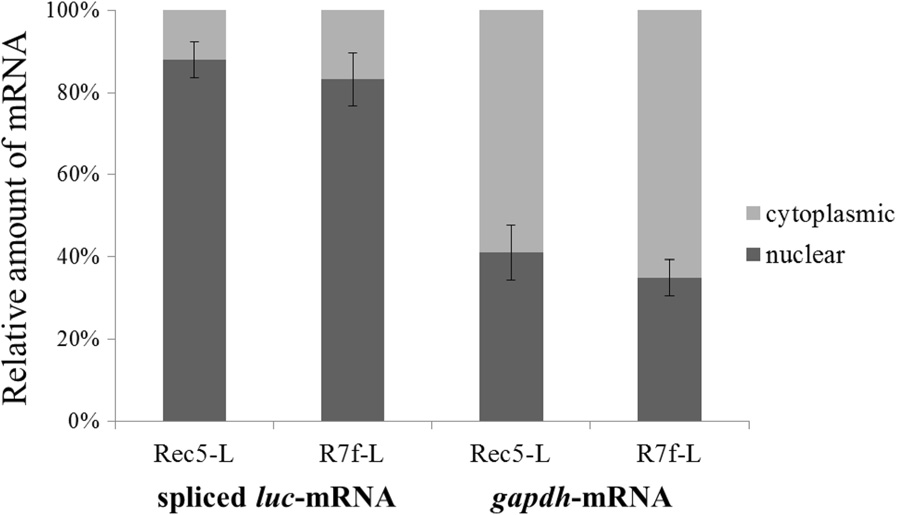
**Nuclear-cytoplasmic distribution of *luc*-mRNA.** Nuclear and cytoplasmic fractions were obtained from NIH3T3 cells transfected with R7f-L and Rec5-L and RNA was extracted from each fraction. The amount of spliced *luc*-mRNA and *gapdh*-mRNA in each fraction was quantified by real-time RT-PCR. The mean values from 3 independent experiments and the SEM are shown. Statistical comparison was done using the *t* test.

Following the results showing that 0.3-kb fragment influenced the amount of spliced messages and subsequently the expression of its corresponding luciferase protein, we set out to determine the nuclear-cytoplasmic distribution of the spliced message. NIH3T3 cells transfected with Rec5-L and R7f-L vectors were divided into nuclear and cytoplasmic fractions and total RNA was extracted from each fraction. The separation of nucleus and cytoplasm was confirmed by assaying for the presence of ribosomal RNAs. The mature 18S and 28S ribosomal RNAs were detected predominantly in the cytoplasmic fraction (data not shown). In the cells transfected with Rec5-L, 12% of *luc*-mRNA was detected in the cytoplasmic fraction and 88% was in the nuclear fraction (Figure 4). In the cells transfected with R7f-L, 17% of *luc*-mRNA was detected in the cytoplasmic fraction and 83% was in the nuclear fraction. In both types of cell, *gapdh*-mRNA was predominantly in the cytoplasmic fraction, with 59% (Rec5-L) and 65% (R7f-L) of the *gapdh*-mRNA in the cytoplasm and about 41% (Rec5-L) and 35% (R7f-L) remaining in the nucleus (Figure 4). The distribution of *luc*-mRNA in the nucleus and cytoplasm of the cells with introduced Rec5-L and R7f-L was not significantly different.

### Point mutation analysis

**Figure 5:**
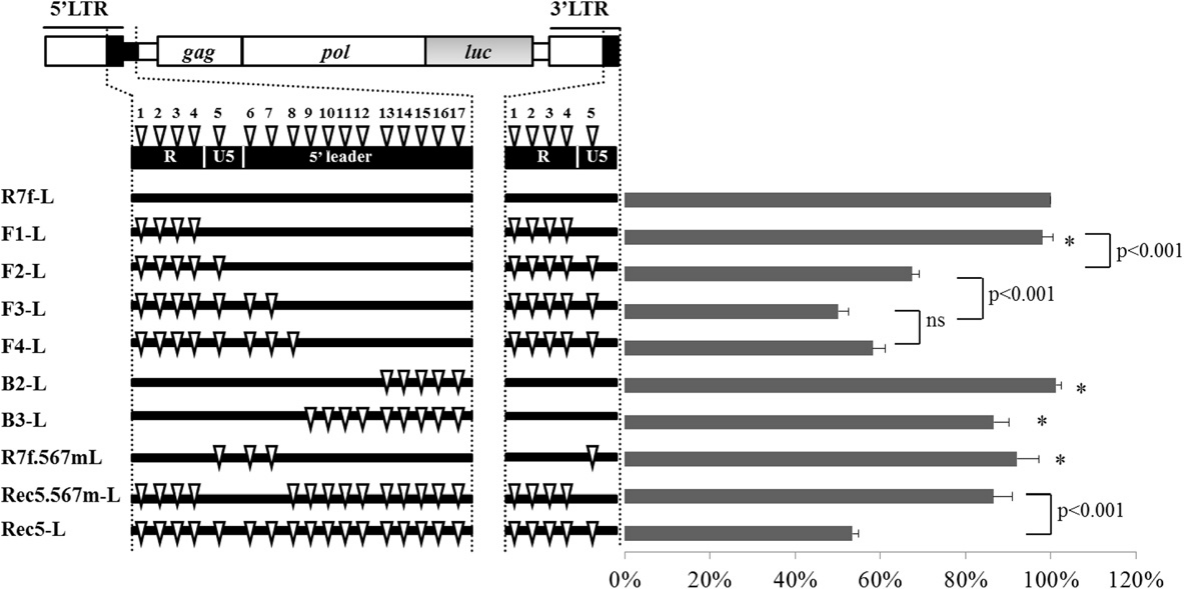
**Luciferase activity of mutation series vectors.** A series of vectors where the sequences from A8 were gradually mutated into 57 sequences were constructed and their luciferase activities were quantified. Mutations from A8 to 57 are indicated by triangles. The mean values from 4–7 independent experiments and the SEM are shown. Statistical comparison was done using the *t* test. ns: differences were not significant. *: differences were not significant versus R7f-L.

Further investigations were carried out to determine whether any of the nucleotides within the 0.3-kb fragment are key(s) to the observed luciferase expression effects. Using the same luciferase expression vectors, a series of point mutations was incorporated into the R7f-L 0.3-kb fragment, in which the 17 nucleotides that differ between the A8 and 57 sequences were gradually mutated into sequences of 57 from the 5’ site. The luciferase activity of these vectors was determined (Figure 5). F1-L, which has its first four nucleotides exchanged for 57 sequences, showed results comparable to those obtained for R7f-L. Interestingly, when further mutations were introduced at the 5th nucleotide in F2-L, the luciferase activity decreased to 67% (p < 0.001) compared to F1-L. The luciferase activity of F3-L, in which further mutations were introduced at the 6th and 7th nucleotides, decreased to 50% and was lower than that of F2-L (p < 0.001). The luciferase activity of F4-L, in which further mutations were introduced at the 8th nucleotide, was the same as that of F3-L. On the other hand, we constructed the B series vectors in which mutations were incorporated from the 3’ site of 0.3-kb fragment. When the 9th to 14th nucleotides were further exchanged for their 57 counterparts, the luciferase activity showed no significant difference compared to R7f-L in B2-L (101%) and B3-L (87%).

After evaluating the results of experiments with the F series vectors, we asked if the 5th, 6th and 7th nucleotides alone could contribute to the regulation of luciferase activity. Towards this end, we constructed: (a) R7f.567 m-L, in which only the 5th, 6th and 7th nucleotides contain the 57 sequences and (b) another vector having the exact reverse order, Rec5.567 m-L, which has only the 5th, 6th and 7th sequences retained as A8 sequences. The luciferase activity of R7f.567 m-L remained at about 95% and could not be brought down to parallel that of Rec5-L, while its exact reverse vector, Rec5.567 m-L, had a significantly increased luciferase activity (86%) that was higher than that of Rec5-L (p < 0.001).

### Secondary structure analysis

**Figure 6:**
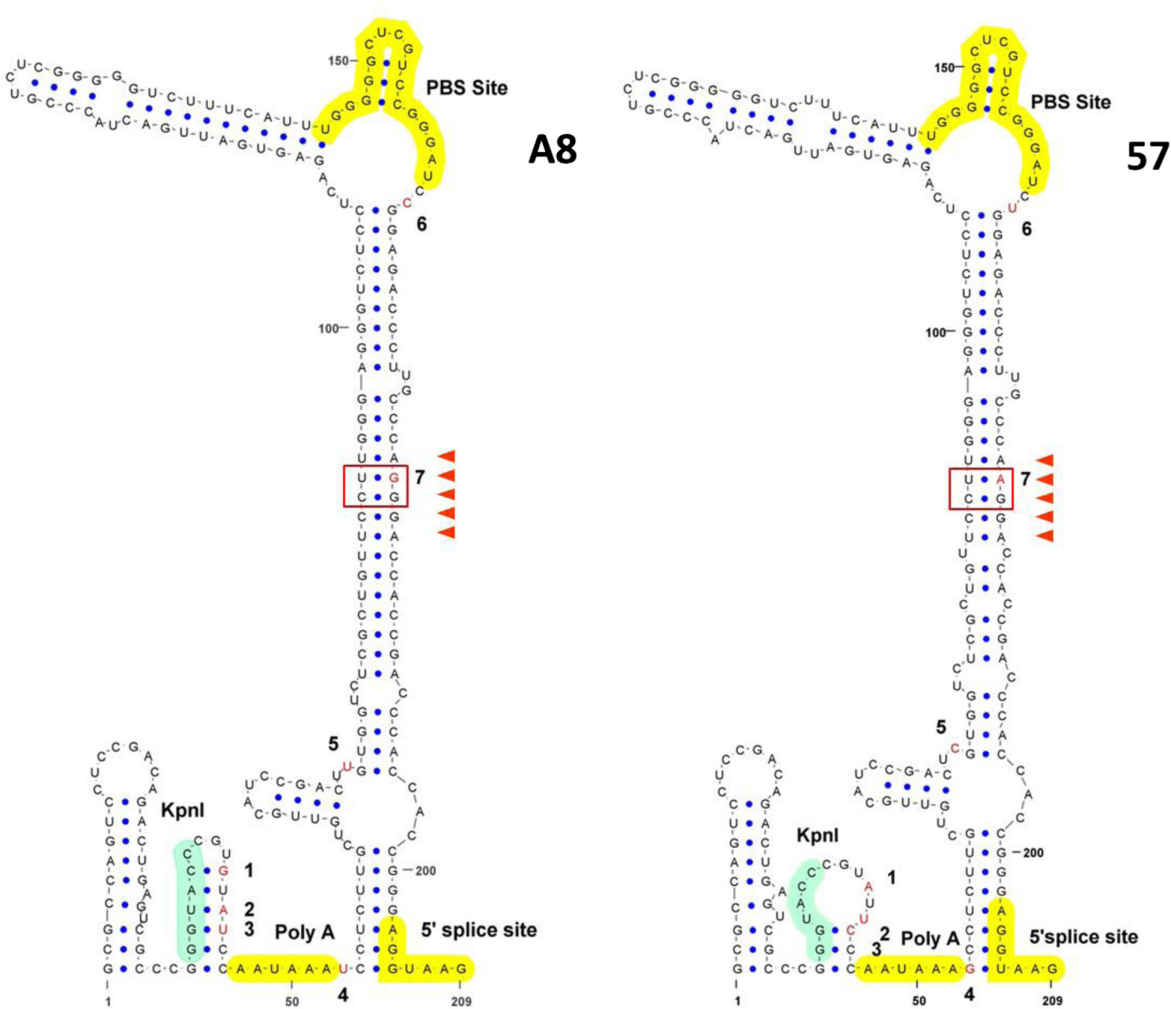
**Predicted secondary structure formed by the sequence containing the 1st to 7th nucleotides of the 0.3-kb fragment of the A8 sequence [accession no. D88386] and the 57 sequence [accession no. X02794].** This representation shows the results of an MFOLD simulation on the basis of previous studies [28,33,41], and the figure was drawn using VARNA software [42]. Nucleotides that differ between A8 and 57 are shown in red and numbered from 1 to 7. Important regulatory signals are highlighted. PolyA: polyadenylation signal; PBS: primer binding site; 5’ss: 5’splice site. Restriction enzyme KpnI site is also shown.

To explain how the 1st to 7th nucleotides might be important for *luc*-mRNA expression, we mapped out the secondary structure formed by the sequence containing the 1st to 7th nucleotides of the 0.3-kb fragment of the A8 and 57 sequences. The secondary structure, as shown in Figure 6, was predicted using MFOLD software. Appropriate regions were selected where the sequence should be truncated by referring to previous studies that had utilized chemical structural probing, NMR, and a functional analysis of Mo-MLV [28,33,41]. Figure 6 illustrates the major functional secondary structures of MLV. At first glance, there is not a striking difference between the two secondary structures generated, despite the 7 nucleotides that differ between the A8 and 57 sequences. The most visible changes actually occur upstream from the polyadenylation signal, where the 1st, 2nd, and 3rd nucleotides are incorporated into a stem structure in the A8 sequence, thereby lengthening the stem structure compared to the 57 sequence. The site with the smallest conformational change contains the 5th, 6th and 7th nucleotides. These three nucleotides reside within a stem-loop structure that protrudes out into the PBS. The possible roles played by these nucleotides are discussed further in the next section.

### Alignment of the 0.3-kb fragment sequences among gamma retroviruses

Since point mutational analysis indicated the 1st to 7th nucleotides contribute to the luciferase activities of the vectors, we compared the sequences including these nucleotides in gamma retroviruses containing MLVs, Feline leukemia virus (FLV), and Gibbon ape leukemia virus (GALV) (Table 1). The 1st guanine (G) nucleotide in A8 was well conserved among these gamma retroviruses except for 57. The 2nd and 3rd nucleotides in A8 were adenine (A) and thymidine (T), respectively, and the 4th, 5th, and 6th nucleotides in 57 were G, cytosine (C), and T, respectively. These nucleotides were relatively conserved among the gamma retroviruses that were analyzed. The 7th guanine nucleotide in A8 was well conserved in not only MLVs but also in FLV and the GALV. Among the sequences analyzed, only the Fr-MLV clone 57 virus had an adenine at the 7th nucleotide.

**Table 1:** Alignment of the 0.3-kb fragment sequences among the gamma retroviruses

**Name**	**Accession no.**	**1st, 2nd, 3rd, 4th nucleotide**	**5th nucleotide**	**6th nucleotide**	**7th nucelotide**
Murine leukemia virus					
***Exogenous ecotropic***					
Friend clone A8	D88386	CCCGTGTATCCAATAAATCCTCTT	CGACTTGTGGT	GGATCCGGAGA	GCCCAGGGACC
Friend clone 57	X02794	*****A*TC********G******	*****C*****	*****T*****	*****A*****
Friend FB29	NC_001362	************************	*****C*****	*****T*****	***********
Friend PVC211	M93134	************************	***********	***********	***********
Moloney	NC_001501	*****************C******	***********	*****G*****	***********
Moloney ts1-92b	AF462057	*****************C******	***A*C*****	****TT*****	***********
Cas-Br-E	X57540	*****************C******	*****G*****	********G**	*******A***
SRS19-6	AF019230	*******T*********G******	***********	***********	***********
SL3-3	AF169256	*****************G***T**	***A*C*****	*****T*****	***********
RadLV	K03363	*****************G***T**	***A*C*****	****TT*****	***********
Rauscher	NC_001819	************************	***********	*****T*****	***********
Graffi GV-1.2	AB187565	*****TCC*--******G******	***A*C****A	***********	***********
***Amphotropic***					
1313	AF411814	*****************C******	****GA*****	****TT*****	***********
***Xenotropic***					
DG-75	AF221065	*******TC********G******	***A*C*****	A***TA*****	***********
NZB-9-1	K02730	*******TC********G***T**	***A*C*****	***********	***********
***Endogenous ecotoropic***					
AKV	J01998	*****************G***T**	***A*C*****	****TT*****	***********
pSR3	M87550	*****************G***T**	***A*C*****	*****T*****	***********
BM5eco	AY252102	*****************G***T**	***A*C*****	****TT*****	***********
***Polytropic***					
MCF1233	U13766	*******TC********G***T**	***AAC****C	****TT*****	***********
***Unclassified***					
Abelson	NC_001499	*****************C******	***********	*****G*****	***********
Feline leukemia virus	NC_001940	********CG-******C*-****	T****C*****	*****GA**CC	C***C******
Gibbon ape leukemia virus	NC_001885	*******G*********A******	G**GCC*****	*****G*A*AC	C*--*******

Alignment of sequences around the 1st to 7th nucleotides (underlined). Asterisks represent sequence identities.

## Discussion

In the present study, to investigate the role of a 0.3-kb KpnI-AatII fragment containing the R-U5-5’leader sequence, recombinant luciferase vectors were constructed by replacing the viral-*env*-gene with the *luc-*gene in proviral sequences to produce R7f-L and Rec5-L (Figure 2A). As shown in Figure 2B, R7f-L exhibited about 2 times higher luciferase expression compared to Rec5-L. This result agrees well with experiments that utilized the chimeric viruses R7f and Rec5, in which the Env protein expression level of R7f-infected cells was higher than that of Rec5-infected cells [5]. Therefore, the experimental system using R7f-L and Rec5-L vectors is useful to analyze the function of the 0.3-kb fragment in Env protein expression. Next, to examine whether the 0.3-kb fragment functions in the 5’LTR-leader sequence and/or in the 3’LTR, we constructed R7fa-L and R7fb-L. R7fa-L contains the 0.3-kb fragment of A8 sequences only at the 5’LTR, and R7fb-L contains the 57 sequences at the 5’LTR but has the A8 sequences of the R-U5 region at the 3’LTR (Figure 2A). The results of a luciferase assay showed that R7fa-L mimics the expression level of R7f-L (Figure 2B). R7fb-L, despite having partial A8 sequences at its 3’LTR, had a similarly reduced expression level of Rec5-L. These results suggested that luciferase expression is dependent solely on the 0.3-kb sequences at the 5’LTR-leader sequence rather than the sequences at the 3’LTR.

In the luciferase expression vector system of the present study, luciferase protein is translated from spliced mRNA. When quantified in transfected cells, the amount of spliced *luc*-mRNA in the cells transfected with R7f-L was about 2 times higher than that in the cells transfected with Rec5-L (Figure 2C). Furthermore, the amount of spliced *luc*-mRNA of R7fa-L was equivalent to the amount of spliced *luc*-mRNA of R7f-L, and R7fb-L showed the same amount of spliced *luc*-mRNA as Rec5-L (Figure 2C). The amount of spliced transcripts from the vectors correlated with the luciferase activities (Figure 2B). These results indicated that the 0.3-kb fragment of A8 enhanced luciferase expression levels by increasing the amount of spliced *luc*-mRNA. This raised the question of how the 0.3-kb fragment of A8 enhanced the amount of spliced *luc*-mRNA. Because the amount of total transcripts, including unspliced mRNA and spliced mRNA, was the same among Rec5-L, R7f-L, R7fa-L, and R7fb-L (Figure 2B), the 0.3-kb fragment seems to not affect the transcriptional step. Other steps in the maturation of transcripts were also investigated, e.g. the poly (A) tail length and the nuclear export of transcripts from vectors. We could not observe any differences between the poly (A) tail length of mRNA in the R7f-L versus the Rec5-L transfected cells (Figure 3). The nuclear-cytoplasmic distribution of spliced *luc*-mRNA was the same for the R7f-L and the Rec5-L transfected cells (Figure 4), indicating that the efficiency of nuclear export of spliced *luc*-mRNA was the same for both R7f-L and Rec5-L. These results suggest that the 0.3-kb fragment contributes to the splicing efficiency of transcripts and that luciferase expression is enhanced by the role of the 0.3-kb fragment of A8 in promoting splicing. As shown in Figure 3, the poly (A) tail length of viral mRNA was longer than that of *gapdh*-mRNA. The reason for this phenomenon is not clear, but release of poly (A) polymerase from viral mRNA might be suppressed. The nuclear-cytoplasmic distribution of mRNA also differs between viral mRNA and *gapdh*-mRNA. It is generally known that nuclear export of mRNA is mediated by multiple protein factors that couple steps of nuclear pre-mRNA biogenesis to mRNA transport [43] therefore, different factors might be recruited in viral mRNA compared to *gapdh*-mRNA.

Next, to investigate the roles of nucleotides that differ between A8 and 57 within the 0.3-kb fragment, we gradually mutated the 17 nucleotides that differ between them and tested their respective luciferase activities. Among the vectors investigated, only the F3-L, which carries the 1st to 7th nucleotides of 57 on the background of the A8 sequence, showed decreased luciferase activity that paralleled that of Rec5-L, which has the 57 sequence (Figure 5). Furthermore, R7f.567 m-L, which has only the 5th, 6th and 7th sequences retained as 57 sequences, showed luciferase activity that remained at about 95% and could not be brought down to parallel that of Rec5-L. These results suggested that the 1st to 7th nucleotide of the 0.3-kb fragment were important regulators of the luciferase protein expression level.

To illustrate how the 1st to 7th nucleotides of the 0.3-kb fragment may be functionally important, a secondary structure was drawn for the fragment containing the 1st to 7th nucleotides of the A8 and 57 sequences, as shown in Figure 6. The 5th, 6th and 7th nucleotides, which mutational analysis had shown were primary contributors to increased luciferase expression, reside within a stem-loop structure that protrudes out into the PBS. It was previously reported that sequences upstream of the 5’ss negatively regulate the splicing of MLV by forming a secondary structure [32]. Kraunus et al. argue that the stem structure plays a role upstream of the 5’ss in determining the accessibility for cellular splice regulators. According to Zychlinski et al., the stem structure or region surrounding the 5’ss regulates the splice donor to be accessed by U1snRNA, thereby regulating MLV splicing [44]. The stability and integrity of the stem-loop structure containing PBS is important to determine the splicing efficiency: higher stability of the stem-loop structure seems to inhibit splicing more efficiently. Similarly, in HIV type 1, it has been reported that the stable hairpin-structure of RNA containing the major 5’ss suppresses the activity of the 5’ss [45]. Interestingly, as shown in Figure 6, the 4th to 7th nucleotides take part in the formation of secondary structure around the 5’ss. Because the secondary structure formed by A8 releases free energy of dG = −72.5 kcal/mol, while 57 releases dG = −75.1 kcal/mol, the stem structure of the 57 sequence is likely more stable than the A8 sequence. This suggests that the stem structure of the 57 sequence inhibits splicing more efficiently than the stem structure of the A8 sequence, resulting in decreased luciferase activity. Kraunus et al. have studied the AGGGA motif in the stem structure, which is a potential binding motif for hnRNPA1, a splice repressor. The results of experiments in which the AGGGA motif was mutated have shown that this sequence contributes to splicing efficiency through altering the secondary structure stability rather than the sequence motif. The AGGGA motif in the A8 sequence is also found around the 7th nucleotide, as shown by arrowheads in Figure 6. This motif may be demolished by changing the A8-G sequence at the 7th nucleotide of 57 to adenine, which may decrease the binding of hnRNPA1, the splice repressor; however, contrary to expectations, luciferase expression was decreased. In examining the secondary structure, the base corresponding to the 7th G on the ascending side of the stem is U in the A8 sequence, while the base corresponding to the 7th A on the ascending side of the stem is U in the 57 sequence (Figure 6, boxed motif). Kraunus et al. reported that the higher complementarity of bases facing each other in the boxed motif decreased the splicing efficiency. This suggests that the 7th nucleotide plays an important role in luciferase expression by participating in the splicing step. Alignment of the gamma retroviral 0.3-kb fragment sequences showed that the A8-guanine at the 7th position is conserved among the FLV, GALV, and MLV sequences except for 57, while the A8-thymine and A8-cytosine at the 5th and 6th positions, respectively, are less conserved. The 7th nucleotide is likely to be important for gene expression of gamma retroviruses, which might explain the different activities of the 0.3-kb fragments of A8 and 57. The roles of the 1st to 4th nucleotides are not yet known, but a change of secondary structure between A8 and 57 has been observed (Figure 6) and this stem loop structure may also contribute to luciferase expression through tertiary interactions with the stem loop structure formed by the sequence containing the 5th to 7th nucleotides.

## Conclusion

In summary, we have described the role of the 0.3-kb fragment containing the R-U5-5’leader sequence of Fr-MLV in gene expression. The 0.3-kb fragment influenced the protein expression level from spliced-mRNA by regulating the efficiency of splicing, rather than transcription, poly (A) addition to mRNA, or nuclear export of spliced-mRNA. Furthermore, seven nucleotides that apparently contribute to regulation of gene expression have been identified. Interestingly, these nucleotides reside within the stem-loop structure that has been speculated to limit recognition of the 5’ss.

## Materials and methods

### Vector construction

Luciferase expression vectors R7f-L and Rec5-L were constructed as described previously by replacing the viral *env* gene with the *luc* gene [46] within its proviral sequences [5]. The point mutations G to T (2608nt), G to T (2614nt), and G to T (2629nt) were introduced into the *pol* gene of each recombinant plasmid. R7fa-L was constructed by replacing the 57 sequences of KpnI (32) and AatII (361) with the A8 sequences in Rec5-L. R7fb-L was generated by replacing the A8 sequences of KpnI (32) and AatII (361) with the 57 sequences in R7f-L. Mutation vector F1-L was constructed by mutagenesis of R7f-L using the following forward primer: CGCCCGGGTACCCGTATTCCCAATAAAGCCTCTTGCTG; and the reverse primer: ACGGGTACCCGGGCGACTCAGTCTA. F2-L was generated by mutagenesis of F1-L using the forward primer: TCTTGCTGTTGCATCCGACTCGTGGTCTCGCTGTT; and the reverse primer: AGTCGGATGCAACAGCAAGAGGCTTTATTG. F3-L was constructed by mutagenesis of F2-L using the forward primer: TTTGGGGGCTCGTCCGGGATCTGGAGACCCTTGCCCAAGGACCACCGA; and the reverse primer: GATCCCGGACGAGCCCCCAAATGAAAGACCC. F4-L was generated by mutagenesis of F3-L using the forward primer: AAGCTGGCCAGCAATTGATCtGTGTCTGTCC; and the reverse primer: GATCAATTGCTGGCCAGCTTACCTCCCGGT. B1-L was generated by mutagenesis of R7f-L using the forward primer: ACCCGTGGTAGAACTGACGGGTTCGAGACACCCGGCCGCAA; and the reverse primer: CGTCAGTTCTACCACGGGTCCGCCAGATA. B2-L was generated by mutagenesis of B1-L using the forward primer: TTGGCCGACTAGCTCTGTACCTGGCGGACCCGTGGTGGAACTGACG; and the reverse primer TACAGAGCTAGTCGGCCAACTAGTACAGAC. B3-L was generated by mutagenesis of B2-L using the forward primer: CCATTGTCCCGTGTCTTTGATTGATTTTATGCGCCTGCGTTTGTACTAGT; and the reverse primer: TCAAAGACACGGGACAATGGACAGACACCG. R7f.5 m-L was constructed by mutagenesis of R7f-L using the forward primer: TCTTGCTGTTGCATCCGACTCGTGGTCTCGCTGTT; and the reverse primer: AGTCGGATGCAACAGCAAGAGGATTTATTG. R7f.567m-L was constructed by mutagenesis of R7f.5m-L using the forward primer: TTTGGGGGCTCGTCCGGGATCTGGAGACCCTTGCCCAAGGACCACCGA; and the reverse primer: GATCCCGGACGAGCCCCCAAATGAAAGACCC. Rec5.5m-L was constructed by mutagenesis of Rec5-L using the forward primer: TCTTGCTGTTGCATCCGACTTGTGGTCTCGCTGTT; and the reverse primer: AGTCGGATGCAACAGCAAGAGGCTTTATTG. Rec5.567m-L was constructed by mutagenesis of Rec5.5m-L using the forward primer: GGAGACCCTTGCCCAGGGACCACCGACC; and the reverse primer: AAGGGTCTCCGGATCCCGGACGAGCCC. Structures of the expression vectors were confirmed by digestion with restriction enzymes and sequence analysis. Basic recombinant DNA procedures were performed according to standard protocols [47].

### Cell culture

NIH3T3 cells were grown in Dulbecco’s Modified Eagle Medium – low glucose (SIGMA) supplemented with 10% fetal calf serum (MP Biomedicals) and penicillin-streptomycin (GIBCO) and cells were incubated at 37°C in a 7% CO_2_ atmosphere. HeLa cells were grown under the same conditions as NIH3T3 except they were incubated in a 5% CO_2_ atmosphere.

### Transfection and assay for luciferase activity

NIH3T3 cells (1 × 10^5^) were plated in 24-well plates with growth medium minus penicillin and transfected the next day with 0.8 ug luciferase expression vectors, 5 ng of pRL-SV40 (Promega) using 2 ul of Lipofectamine 2000 Reagent (Invitrogen, Carlsbad, CA, USA) diluted with OPTI-MEM (Invitrogen). After 48 hours, cells were lysed and luciferase activities were measured as Relative Light Units (RLU) using a luminometer with a Dual-Luciferase Reporter Assay System (Promega) according to the manufacturer’s instructions. The luciferase activity of each sample was normalized to that of Renilla (pRL-SV40) as an internal control.

### RNA extraction and quantification

RNA extraction was carried out using an RNase Mini Kit (Qiagen). RNA was treated with RNase-free DNase (Qiagen) and 2 ug of RNA were reverse transcribed using an OligodT_20_ primer and SSIII reverse transcribing kit (Invitrogen). A portion of the resulting cDNA was subjected to real-time PCR using an Applied Biosystems® 7500 Real-Time PCR System. The specific primers and probes used for detection of total mRNA at the *luc* region were: LucF: CGGCTTCGGCATGTTCA; LucR: TACATGAGCACGACCCGAAA: TaqMan probe: CACGCTGGGCTACTTGATCTGCGG. *Spliced*-mRNA was detected using s6: GGGTCTTT CATTTGGGGGCTC; s2: TGCCGCCAACGGTCTCC and the TaqMan probe: CACCACCGGGAGCTCATTTACAGGCAC. Standard curves to quantify both mRNAs derived from the luciferase expression vectors utilized vector splA8L [46]. In addition, *gapdh*-mRNA was quantified as an internal control using TaqMan Rodent GAPDH Control Reagents containing primer sets and probes (Applied Biosystems). Standard curves to calculate the amount of mRNA were created using serially diluted *gapdh* T-easy vector. The negative control samples without the cDNA synthesis step showed undetectable amplification.

### Genomic DNA extraction and quantification

Cellular genomic DNA (gDNA) was extracted using a DNeasy Blood and Tissue Kit (Qiagen) according to the manufacturer’s instructions. Real-time PCR was performed to quantify the amount of plasmid DNAs introduced into the cells. Primers and probe sets used to quantify the amount of firefly luciferase expression vector introduced were the same TaqMan primer and probe set used to detect the amount of cDNA. The amount of *gapdh* DNA was measured as an internal control using the TaqMan Rodent GAPDH Control Reagents.

### Cell fractionation

Nuclear and cytoplasmic fractions were obtained from cultured cells using a PARIS kit (Ambion) according to the manufacturer’s manual. As a control for the fractionation, an aliquot of total RNA from each section was electrophoresed on a 1% agarose gel in morpholinepropane-sulfonic acid (MOPS) buffer, and the cellular ribosomal RNAs were visualized by ethidium-bromide staining.

### Determination of poly (A) tail length

Total RNA extracted from 24 hours post-transfected Hela-cells were ligated with RV3PC–anchor primers. Reverse transcription was then carried out using an antisense sequence of the RV3PC-anchor primer. To amplify the poly (A) tail of mRNA, a forward primer targeting the 3’end of U3 at LTR (AGCTCACAACCCCTCACTCGGC) was paired with a reverse primer targeting the RV3PC-anchor sequence. To increase the likelihood of the reverse primer binding at the poly(A) tail, ten thymines were added into the 3’end of the reverse primer sequence (CTAGCAAAATAGGCTGTCCCTTTTTTTTTT). Likewise, to detect the poly (A) tail length of the *gapdh*-mRNA, a forward primer, Mgapdh3end (CCCTACTCTCTTGAATACCATCA), was set at the junction before the poly(A) signal and was used with the same reverse primer targeting the RV3PC-anchor sequence. The resulting PCR products were stained in ethidium bromide and electrophoresed on an 8% polyacrylamide gel to visualize the spliced mRNA. A 3% agarose gel was used to visualize *gapdh*-mRNA. Within the pool of reverse-transcribed cDNA, the following primers were used to detect the presence of *luc*-mRNA: forward primer f-597 (GGGCTCGTCCGGGATC) and reverse primer s2 (TGCCGCCAACGGTCTCC); for *gapdh*-mRNA, the forward and reverse primers from the Taqman Rodent GAPDH control reagents (Applied Biosystems) were used.

## Competing interests

The authors declare that they have no competing interests.

## Authors’ contributions

YS carried out real-time PCR analyses and luciferase assay experiments. NO constructed some of the vectors and carried out luciferase assays. AM determined the poly (A) tail length. STY conceived and organized the study and helped to draft the manuscript. All authors read and approved the final manuscript.
